# Cats Parallel Great Apes and Corvids in Motor Self-Regulation – Not Brain but Material Size Matters

**DOI:** 10.3389/fpsyg.2018.01995

**Published:** 2018-10-22

**Authors:** Katarzyna Bobrowicz, Mathias Osvath

**Affiliations:** Cognitive Zoology Group, Department of Philosophy and Cognitive Science, Lund University, Lund, Sweden

**Keywords:** behavioral inhibition, domestic cat, cylinder task, detour, motor self-regulation

## Abstract

The inhibition of unproductive motor movements is regarded as a fundamental cognitive mechanism. Recently it has been shown that species with large absolute brain size or high numbers of pallial neurons, like great apes and corvids, show the highest performance on a task purportedly measuring this mechanism: the cylinder task. In this task the subject must detour a perpendicularly oriented transparent cylinder to reach a reward through a side opening, instead of directly reaching for it and bumping into the front, which is regarded as an inhibitory failure. Here we test domestic cats, for the first time, and show that they can reach the same levels as great apes and corvids on this task, despite having much smaller brains. We tested subjects with apparatuses that varied in size (cylinder length and diameter) and material (glass or plastic), and found that subjects performed best on the large cylinders. As numbers of successes decreased significantly when the cylinders were smaller, we conducted additionally two experiments to discern which properties (length of the transparent surface, goal distance from the surface, size of the side opening) affects performance. We conclude that sensorimotor requirements, which differ between species, may have large impact on the results in such seemingly simple and apparently comparable tests. However, we also conclude that cats have comparably high levels of motor self-regulation, despite the differences between tests.

## Introduction

Motor self-regulation overrides unproductive motor movements triggered by salient stimuli in the environment (Beran, 2015). Such behavioral inhibitions are thought of as basic inhibitory abilities within the larger family of executive functions (Diamond, 2013). This mechanism is fundamental in the sense that poor motor self-regulation will lead to difficulties in executing other, more cognitively demanding, tasks. In a sense, without any motor self-regulation one gets stuck in the immediate sensorimotor moment.

This underlying character of motor self-regulation has made it appealing for species comparisons. For example, is there a correlation between these basic skills and performance on other cognitive tasks? A large-scale study by MacLean et al. (2014) comparing 567 individuals from 36 species, found a correlation between (mainly) absolute brain size and motor self-regulatory performance on two tasks: the cylinder task and the A-not-B test (2014). The cylinder task, which was administered to the largest number of subjects and species in the study, consists of a transparent cylinder, containing a reward in the middle, with openings at both ends. To successfully retrieve the reward the subject must refrain from immediately reaching for it through the shortest perceivable route (thereby bumping into the transparent surface) and instead make a detour to either side opening. The best performing taxon on this task was the primates, with great apes reaching top scores. A later study showed that corvids from the genus *Corvus* are as successful as great apes at solving the cylinder task, despite vastly smaller absolute brain sizes (Kabadayi et al., 2016). On the other hand, within birds the corvids have the largest number of pallial neurons and they show cognitive complexity in various studies (Olkowicz et al., 2016).

It is perhaps not surprising that cognitively sophisticated species find tasks like these simple, however, it is noteworthy that many other species fail to such degrees. In other words, why do so many animals show poor performance in motor self-regulation tasks, despite the fact that it appears to be an essential form of inhibition in the complex lives of mammals and birds (e.g., when stalking prey or foraging at great heights)? We hypothesize that the cylinder task may measure additional factors that may not in an immediate sense be related to motor self-regulation, such as the sensorimotor adaptations of different animals.

We investigated the performance of domestic cats (*Felis catus*) on different versions of the cylinder task. Cats arguably benefit from pronounced motor self-regulation due to their reliance on stalking and stealth during hunting, and hence the need to avoid alerting their prey in the wrong moment even when it can be clearly seen by the cat. Cats are also avid climbers and would therefore benefit from motor self-regulation when negotiating narrow supports high above ground. On the other hand, cats have an average mammalian relative brain size, and their absolute brain size is comparably small (Roth and Dicke, 2005; Finarelli, 2006), as is their number of neurons and neuronal density (Jardim-Messeder et al., 2017).

**FIGURE 1:**
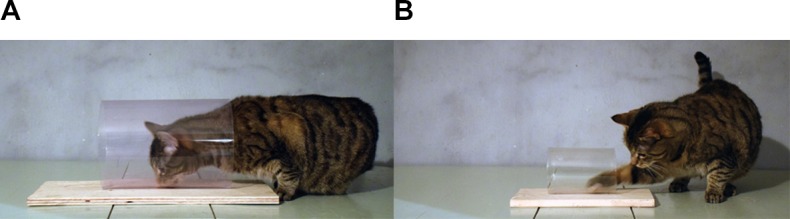
The two different sizes of cylinders used in the study and the retrieval behaviors they afford. **(A)** The large cylinder allows the cat to retrieve the reward directly with its mouth, but also by its paw. **(B)** The small cylinder only allows food retrieval by a paw. Note that both cylinders in the figure are made from plastic.

Retrieving a relatively small item from a cylinder likely requires a certain level of sensorimotor proficiency, e.g., hand-limb coordination and degrees of mobility in relevant joints. To test whether sensorimotor requirements influence performance on the cylinder task, we presented cats with two different cylinder sizes: large or small (Experiment 1). The larger cylinders allowed for more behavioral options such that the cats could use either their head (mouth) or paws to retrieve the reward, and consequently did not require as precise motor planning as the small ones, where the cats were restricted to using a paw (see Figure 1). Moreover, the different sizes resulted in that the reward was positioned at different distances from the transparent surface, i.e., in a large cylinder the surface was further away from the reward. Previous studies have shown that the cats and some other species performed better in similar detour tasks if the reward was partially occluded, e.g., if the barrier was made from a mesh instead of a fully transparent surface (cats: Schiller, 1950, other species: Kabadayi et al., 2017). We therefore presented each cylinder size in two different materials, glass and plastic, which reflect light differently. If the animal perceives the barrier to a greater extent, even if it is transparent, then this might benefit motor self-regulation.

In each condition we largely followed the methods of the recent studies on the cylinder task in order to obtain comparable results (MacLean et al., 2014; Kabadayi et al., 2016).

Thereafter, we conducted two control experiments (Experiment 2 and 3) with the same subjects. The cylinders in Experiment 1 differed in terms of: (1) length of transparent surface, (2) distance between the surface and the reward, and (3) opening size. Experiment 2 and 3 tested which of these factors had the largest influence on the performance in Experiment 1.

## Experiment 1: the Joint Effect of Size and Material

### Materials and Methods

#### Subjects

We tested eight adult cats, three of which were females (see Table 1). Their ages ranged between 2 and 13 years. The cats were kept as pets in two different households (four cats from each household). All subjects lived both in- and outdoors in a rural environment and had daily experience with transparent surfaces, such as windows.

**Table 1:** Individual information for the tested cats.

Name	Sex	Age	Group
**Ina**	Female	4	1
**Filemon**	Male	4	1
**Timmy**	Male	5	1
**Stefan**	Male	8	1
**Neffie**	Female	13	2
**Lupus**	Male	4	2
**Milton**	Male	2	2
**Mira**	Female	9	2


#### Apparatuses

**FIGURE 2:**
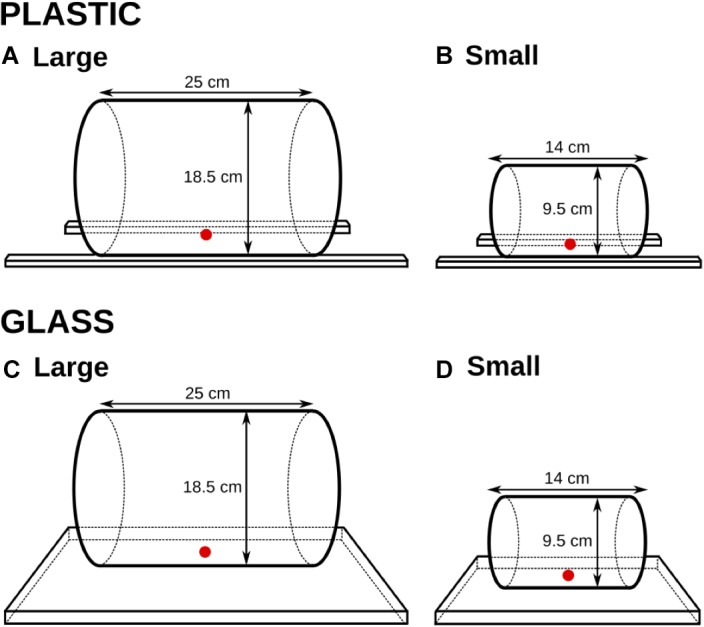
The four cylinders and their dimensions (Experiment 1). The reward was inserted either into a large-size cylinder **(A,C)** or a small-size cylinder **(B,D)**. Two cylinders – one plastic **(A,B)** and one glass **(C,D)** – were manufactured within each size.

We used four different transparent cylinders, and corresponding opaque cylinders, of glass and plastic. Two cylinders – one glass and one plastic – were large enough for the cats to retrieve the food with their mouths (18.5 cm in diameter and 25 cm in length), and two – one glass and one plastic – were small so as to encourage food retrieval by a paw (9.5 cm in diameter and 14 cm in length). See Figures 1, 2.

#### Procedure

Before the subject was tested on a transparent cylinder, it was familiarized with the task on a same-sized opaque cylinder, that is, each subject received familiarization with each size within the respective experiment. The subject always watched the experimenter place the food item inside the cylinder. In the familiarization phase the cat received five trials where it could retrieve the reward from any of the two side openings. The trial started when the cat was approximately one meter from the broad side of the cylinder and the experimenter inserted the reward in the middle of the cylinder. The side from which the reward was inserted was pseudo-randomized. The subject had to perform four correct detours out of five trials before testing commenced on the corresponding size a day later. All subjects completed the trials on the first try.

On the following day the cats were tested on the transparent cylinder. The procedure was identical to the familiarization phase. All cats were first tested on the large glass cylinder (*N* = 10 trials per condition), followed by the small plastic one, and thereafter by the small glass and the large plastic cylinder, with varying time intervals in between (see Supplementary Table 1). The reason for this order was that we hypothesized that the large cylinders would be easier to succeed in, and any carry over effect to the small cylinders would make the comparison more conservative (the number of individuals was too low to make counterbalanced test orders statistically meaningful). A failure was recorded if the cat touched the front of the cylinder with its snout or paw before entering the side opening and retrieving the reward. That is, if the animal oriented toward the visible reward from the front of the cylinder and touched its surface, it failed the trial. Regardless of failures, the subjects were eventually allowed to retrieve the reward. There was no time limit for completing a trial and none of the subjects left before retrieving the reward. The tests were conducted indoors in a well-lit room. The experimenter was familiar to the cats.

#### Statistics

Two-way ANOVA for 2^1^ factorial design was used to estimate the effect of size and material on the success rate. The results were further confirmed in best general linear model selection with the individual score as a response variable, and two predictor variables: size and material. To test for the learning effect between the second and the first half of trials, a paired Wilcoxon signed rank test with continuity correction was used. All statistical analyses were conducted in *R* (v.3.3.2, the R Foundation for Statistical Computing^2^). Significance level was set at 0.05.

## Results

**FIGURE 3:**
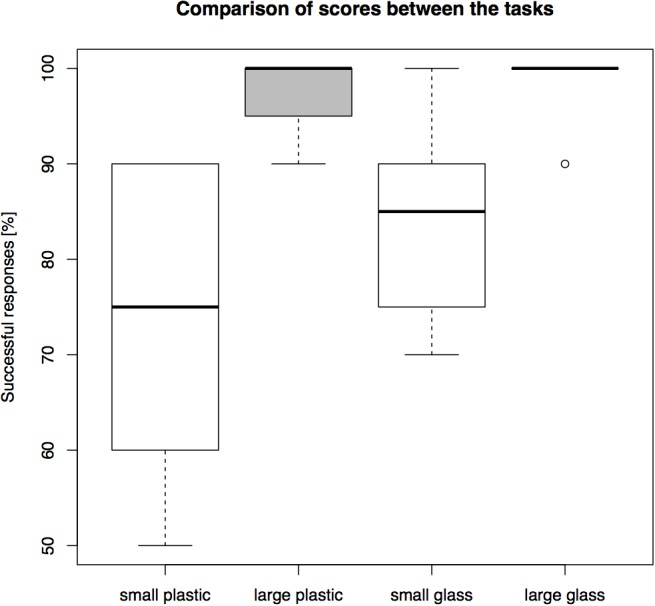
A comparison of overall performance on the four cylinders in Experiment 1 (percentage of *N* = 10 trials correct).

In all failed trials, the cats directly oriented toward the front of the transparent barrier and touched it with its snout or paw, i.e., they did not ever first move toward a side opening. The success rates differed between conditions (see Figure 3 for overall results). Percentages were calculated for each individual and then a group average of these percentages was calculated. The highest average group level of success, 98.75%, was found in the large glass cylinder. This was followed by 97.5% success in the large plastic cylinder. In the small glass cylinder the subjects succeeded in 83.75%, and in the small plastic cylinder they reached 73.75% success.

The main effect of material [*F*(1,32) = 3.982, *p* = 0.056] and the interaction effect [*F*(1,32) = 1.524, *p* = 0.227] on the individual success rates were not significant, while the main effect of size was significant [*F*(1,32) = 22,462, *p* < 0.001] (see Supplementary Figure 1). *Post hoc* comparison using the Bonferroni correction confirmed the significance of the main effect of size on the success rate (*p* < 0.001).

The number of failures did not decrease over trials in any barrier (see Supplementary Table 2). We did not detect a learning curve over trials in any of the four conditions. A paired Wilcoxon signed rank test with continuity correction showed no significant difference between the success rate in the first five and the second five trials in any of the four conditions (small plastic: *p* = 0.1427, small glass: *p* = 0.4568, large plastic: *p* = 1, and large glass: *p* = 1). Overall, the number of failures was highest in the third and the 10th (last) trial (19%), and lowest in the first trial (3%).

## Experiment 2: the Joint Effect of Barrier Length and Goal Distance

### Materials and Methods

#### Subjects

Seven out of eight subjects that participated in Experiment 1 were included in this experiment (Filemon died from unrelated causes after Experiment 1).

#### Apparatuses

**FIGURE 4:**
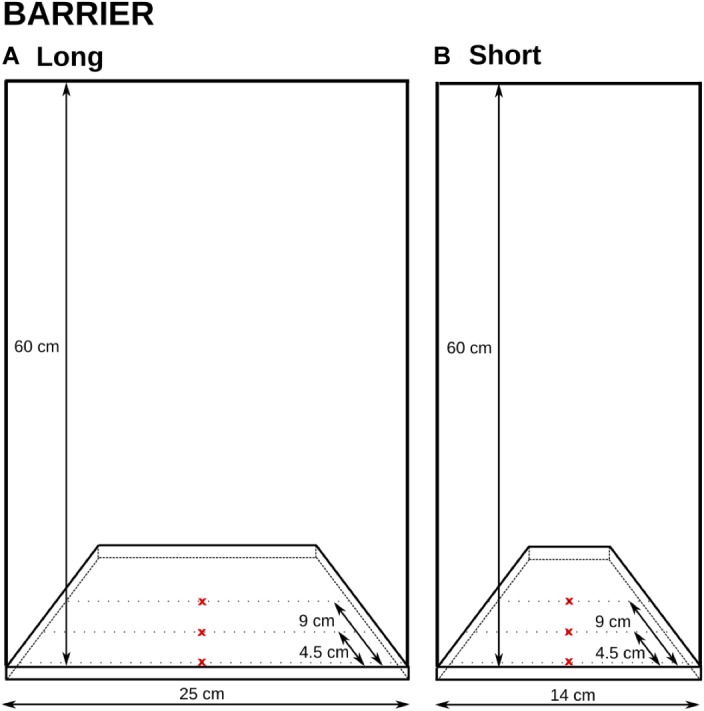
The two setups in Experiment 2 and their dimensions. The reward was placed on one of the three x-marked spots in each condition and was therefore displayed either right behind the transparent surface, or 4.5 cm, or 9 cm from the surface. The barrier length was also manipulated between the trials (**A**: 25 cm; **B**: 14 cm).

Two apparatuses were used, which allowed for barrier length and goal distance manipulation. Both apparatuses consisted of a flat wooden base with a vertical 60 cm-high Plexiglas barrier attached to it; this height was chosen so it would not encourage the subjects to go over it. The length of the barrier mirrored the lengths of the different cylinders in Experiment 1: 25 and 14 cm (see Figure 4).

Three distances from the barrier were used for the reward presentation. The reward was always placed in the middle (equidistant from both end sides). Two distances mirrored the distances to the rewards in Experiment 1: 4.5 cm (the small cylinders) and 9 cm (the large cylinders). An additional distance of 0 cm (touching the barrier) was also added to gain additional data on the potential importance of distances to rewards behind transparent surfaces.

#### Procedure

Experiment 2 was not preceded by a familiarization phase as all subject already had experience of detouring transparent surfaces. All cats were tested 10 times on each of the six sub-conditions (distance of 0 or 4.5 or 9 cm × barrier length of 14 or 25 cm), in total 60 trials each. The cats received up to 20 trials per day (see Supplementary Table 3), and the order of trials was pseudo randomized. Again, a failure was recorded if the cat touched the front surface with its snout or paw before retrieving the reward (through a detour on either side), the tests were conducted with the same surrounding conditions and with the same experimenters as in the previous experiment.

#### Statistics

Two-way ANOVA for 2^3^ factorial design to estimate the effect of barrier length and goal distance on the success rate. The results were further confirmed in best general linear model selection with the individual score as a response variable, and two predictor variables: barrier length and goal distance. To test for the learning effect between the second and the first half of trials, a paired Wilcoxon signed rank test with continuity correction was used. All statistical analyses were conducted in *R* (v.3.3.2, the R Foundation for Statistical Computing^2^). Significance level was set at 0.05.

## Results

**FIGURE 5:**
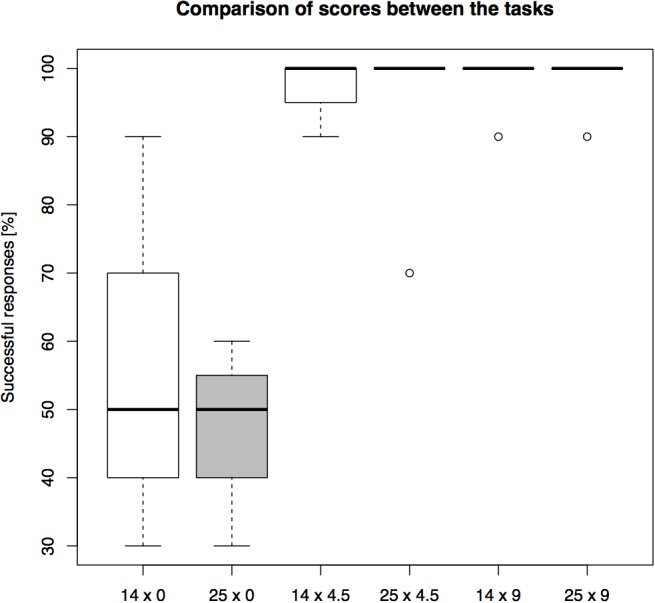
A comparison of overall performance on the setups in Experiment 2. The barrier length (14 or 25 cm) and the goal distance (0 or 4.5 or 9 cm) were manipulated between the trials (percentage of *N* = 10 trials correct).

The success rates differed between conditions (see Figure 5 for overall results). The highest average group level of success, 98.57%, was found in the 9 cm distances, regardless of the barrier length. This was followed by 97.14% success in 4.5 cm distance in the short-barrier (14 cm), and 95.71% success in the 4.5 cm distance in the long-barrier (25 cm). The lowest success rate was scored in the 0 cm distance: 62.86% in the short-barrier, and 47.14% in the long-barrier condition. Therefore, the effect of the barrier length on overall performance – expressed in the difference between respective success rates in the short-barrier and the long-barrier – increased with the decrease in the goal distance (0% in the 9 cm distances, 1.43% in the 4.5 cm distances, and 15.72% in the 0 cm distance).

However, the main effect of barrier length [*F*(1,42) = 1.851, *p* = 0.182] and the interaction effect [*F*(2,42) = 0.531, *p* = 0.592] on the individual success rates were not statistically significant, while the main effect of goal distance was significant [*F*(2,42) = 29.863, *p* < 0.001] (see Supplementary Figure 2). *Post hoc* comparison using TukeyHSD test revealed significant differences between the “0 cm distance” and the “4.5 cm distance” conditions (*p* < 0.001), and the “0 cm distance” and the “9 cm distance” conditions (*p* < 0.001). However, there was no significant difference between 4.5 and 9 cm (*p* = 0.881).

We detected a learning signature effect over trials in only one of the six conditions: the 0 cm distance in long-barrier condition. A paired Wilcoxon signed rank test with continuity correction showed a significant difference between the success rate in the first five and the second five trials (*p* = 0.034). For more details see Supplementary Table 4.

## Experiment 3: the Joint Effect of Barrier Length and Opening Size

### Materials and Methods

#### Subjects

The same subjects were tested as in Experiment 2.

#### Apparatuses

**FIGURE 6:**
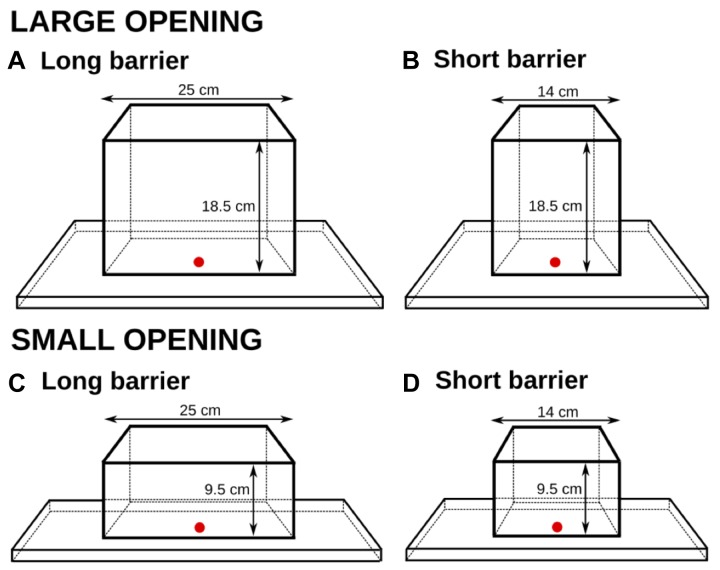
The four plastic cuboids in Experiment 3 and their dimensions. The goal distance was the same for all cuboids; the barrier length (**A,C**: 25 cm; **B,D**: 14 cm) and the opening size (**A,B**: large; **C,D**: small) were manipulated.

Four different transparent cuboids were used to manipulate the barrier length and the opening size. All cuboids were made of Plexiglas and differed in two parameters: the length of the transparent surface (14 cm vs. 25 cm, again mirroring the lengths of the cylinders in Experiment 1), and the width of the square side opening (18.5 cm vs. 9.5 cm, which mirrored the diameters in the large and the small cylinders in Experiment 1). The distance to the reward behind the surface was constantly kept at 4.5 cm (see Figure 6).

#### Procedure

No familiarization phase preceded this experiment due to the cats’ previous experience. All cats were tested in ten trials on each of the four sub-conditions (opening width of 18.5 cm or 9 cm × barrier length of 14 or 25 cm), executing a total of 40 trials each. The cats received up to 20 trials per day (see Supplementary Table 5), and the order of trials was pseudo randomized. A failure was recorded as in previous experiments. The tests were conducted during the same conditions and with the same experimenters as in previous experiments.

#### Statistics

Two-way ANOVA for 2^2^ factorial design to estimate the effect of barrier length and opening size on the success rate. The results were further confirmed in best general linear model selection with the individual score as a response variable, and two predictor variables: barrier length and opening size. To test for the learning effect between the second and the first half of trials, a paired Wilcoxon signed rank test with continuity correction was used. All statistical analyses were conducted in *R* (v.3.3.2, the R Foundation for Statistical Computing^3^). Significance level was set at 0.05.

## Results

**FIGURE 7:**
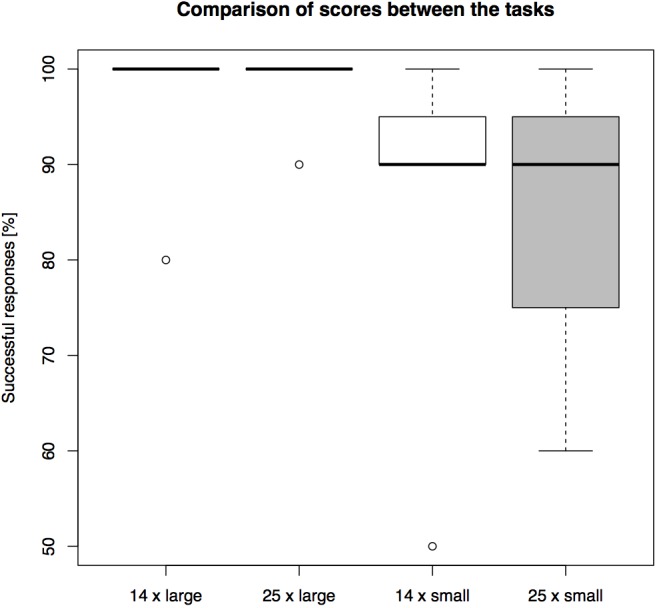
A comparison of overall performance on the setups in Experiment 3. The barrier length (14 or 25 cm) and the opening size (large or small) were manipulated between the trials (percentage of *N* = 10 trials correct).

The success rates differed between conditions (see Figure 7 for overall results). The highest average group level of success, 98.57%, was found in the “long-barrier large-opening” condition, followed by 97.14% success in the “short-barrier large-opening condition”. In the two length conditions with the smaller openings the subjects reached 87.14% success on the short barrier and 84.29% success on the long barrier.

Again, the main effect of barrier length [*F*(1,27) = 0.024, *p* = 0.878] and the interaction effect [*F*(1,27) = 0.218, *p* = 0.645] on the individual success rates were not significant, but the main effect of opening size was significant [*F*(1,27) = 6.992, *p* = 0.014] (See Supplementary Figure 3). A paired Wilcoxon signed rank test with continuity correction showed no significant difference between the success rate in the first five and the second five trials in any of the four conditions (see Supplementary Table 6).

## Discussion

Overall, the cats’ performance was affected by the parameters of the task: distance from the barrier to the reward and the size of the side opening of the apparatus, regardless of its shape. Both 9.5 and 18.5 cm distances between the barrier and the reward– as opposed to no distance – increased the successes. Larger side openings did also increase the number of successful trials.

The performance of the cats in the large cylinders paralleled that of great apes and corvids. In their first task – the large glass cylinder – they were successful on 98.75% of trials, which is surpassed only by chimpanzees, orangutans, and ravens, but better than bonobos, gorillas, jackdaws, and New Caledonian crows (MacLean et al., 2014; Kabadayi et al., 2016). This relationship holds true also for the second cylinder they were tested on, the large plastic one (97.5%). Their performance on the small cylinders tells a different story. In the small glass cylinder (83.75%) they would end up as number ten among best performers of the so far 40 tested species, outperforming e.g., macaques, dogs and wolves. However, when they are given a small plastic cylinder (73.75%) they are well outside top ten, and notably worse than macaques, dogs, wolfs, and scrub jays.

From this we can conclude that caution is needed when making large-scale comparisons including correlations with brain sizes and other factors. If the same eight cats can differ this much between different sizes and materials it becomes difficult to discern which one is the appropriate comparable measure. Importantly, there are no visible learning effects that could explain the differences; on the contrary, success appears to decrease over conditions, which appears due to the size of the cylinders.

The difference in performance between materials was not significant, although it should be noted how much the differences in the small cylinders affect this species’ “ranking” in relation to other species. It does not appear that the differences in how the used materials reflect light affected performance, however, it could be that other materials – such as mesh, which to a larger extent occludes the reward – might improve performance in the smaller cylinders.

As we hypothesized, the larger sizes may require less motor planning; that is, not having to settle for a very specific solution (i.e., use the paw and a stretching motion) but instead allowing subjects to use a behavior that likely has been used more often in relation to food retrieval. We indeed found an effect of the opening size on the subjects’ performance, with higher success rates on the large than on the small openings, regardless of the barrier length. Being able to follow through a motor plan instead of reaching toward the visible but unattainable food is of course a type of motor self-regulation. Should this hypothesis be true, however, it is difficult to know what the animal has to inhibit. For example, a member of one species with a certain sensorimotor adaptation might have to inhibit less than a member of a different species, so better performance in the exact same task might not reveal higher levels of motor self-regulation. Furthermore, animals of some species may be simply more proficient at planning and executing detours with body/limbs to retrieve small items from within constrained spaces than others. This is also true for animals of the same species, as some might have less motor flexibility than others.

Another possibility is that the distance between the transparent surface and the reward influences performance. If the barrier is closer to the animal, i.e., that the reward is further away on the other side, it could trigger a stronger inhibitory response, either because the movement cannot be extended as much as it would be closer before bumping into the surface, or because the animal can keep the reward better in sight through the whole detour. In fact, it has been shown in other detour tasks that a longer distance to the reward produces better results (Poucet et al., 1983). In the current study, we also found an effect of distance. However, as there was no significant difference between the 4 cm and the 9 cm distances – which mirrored the distances in the original cylinders – the distances probably played little role for their performance. It is worth noting however, that a higher effect was found in the 0 cm distance, and that the effect was most prominent when the barrier was long – even accompanied with a learning signature effect, which was not found in other conditions. So, even if it is unlikely that the distance to the reward affected the cats’ performances in Experiment 1, distance might still be an important factor to take into account when comparing species of different sensorimotor compositions and sizes. However, caution is needed in such comparisons as well. Even if the effect of distance may hint on levels of behavioral inhibition of an animal, it can also be an effect of animal’s visual system, e.g., its depth perception from a close distance. Cats are visually oriented in their foraging and have a well-developed stereoscopic vision (Kang et al., 2009), which made them good candidates for comparison to apes and corvids on the cylinder task. However, they have somewhat poorer vision for near distances (where they compensate with vibrissae) as compared to medium-distances (Fitzgerald, 1940; Ahl, 1986), which might explain the cats’ low performance in the 0 cm distance.

Although it was previously reported that the length of a transparent barrier could affect cats’ performance in another detour task (Poucet et al., 1983), we did not detect such an effect. Moreover, the success rate did not increase over trials in any of the conditions, which has previously been reported for cats, as well as for some other species (Schiller, 1950; Kabadayi et al., 2017).

Our findings do not necessarily argue against the hypothesis that animals with the largest brains, or most cortical or pallial neurons, are better in tasks like these; their performance might be good regardless of the cylinder size or material. However, even for them differences might evoke various types or magnitudes of inhibitory responses. In other words, to ensure that one is comparing the same skills, one should consider sensorimotor differences, such as the visual system and detailed motor abilities, and, if possible, adjust the task to accommodate such differences. A common way to investigate a skill is to administer different types of tasks that would each purportedly measure it, as was done in the above-mentioned large-scale study. However, we believe that even more precise measurements could be achieved by also adding variation to the very same tasks in respect to sensorimotor requirements. By doing this with the cylinder task we suggest that the motor self-regulation skills of cats are well developed, reaching a mean of 88.4% of the combined results. Admittedly however, there are not yet any other species to compare within this set-up.

## Data Availability

All datasets generated for this study are included in the manuscript and the supplementary files.

## Ethics Statement

The study did not require any ethical approvals according to Swedish legislation as the research was conducted on privately owned animals and was purely appetitive and non-invasive. Informed consent has been granted by the owners.

## Author Contributions

Both authors contributed equally and agreed to be held accountable for the contents of this work and approved the final version of the manuscript.

## Conflict of Interest Statement

The authors declare that the research was conducted in the absence of any commercial or financial relationships that could be construed as a potential conflict of interest.

## Footnotes

1 http://www.R-project.org

2 http://www.R-project.org

3 http://www.R-project.org
