# Supplementary material for: Cats Parallel Great Apes and Corvids in Motor Self-Regulation – Not Brain but Material Size Matters
Source: Front Psychol. 2018 Oct 22;9:1995. doi: 10.3389/fpsyg.2018.01995 (PMC6204371; doi:10.3389/fpsyg.2018.01995)
Supplement: Supplementary file 1 [file Data_Sheet_1.docx]

Supplementary Material

Cats Parallel Great Apes and Corvids in Behavioral Inhibition
but Size Matters

Katarzyna Bobrowicz*, Mathias Osvath

*** Correspondence:** Katarzyna Bobrowicz: katarzyna.bobrowicz@lucs.lu.se

# Experiment 1: Procedure

For details on the timing of conditions see Supplementary Table 1.

**Supplementary Table 1**. The time elapsed (in days) since the first condition in Experiment 1.

| **Group** | **Condition** | | | |
| --- | --- | --- | --- | --- |
|  | **Large glass** | **Small plastic** | **Small glass** | **Large plastic** |
| **1** | 0 | 133 | 140 | 142 |
| **2** | 0 | 44 | 74 | 76 |

# Experiment 1: Results

We used the analysis of variance for 2^2^ factorial design to estimate the effect of size and material on the success rate and confirmed the results in the best general linear model selection with the individual score as a response variable, and two predictor variables: size and material. The analysis revealed neither the interaction effect nor the main effect for material (*F*(1) = 2.749, *p* = 0.09734). The main effect for size was significant (*F*(1) = 34.123, *p* = .000000005174). For details see Supplementary Figure 2.

**Supplementary Figure 1:** Main effects for size and material of the cylinders on subjects’ average success rate (percentage of N=10 trials correct).

We tested two populations (two households), with four cats each. A one-way between-subjects ANOVA revealed no significant difference in the success rate between these populations, both for the overall performance, and for each condition separately **(**small plastic: F(1,78)=1.61, p=0.208; small glass: F(1,78)=0.09, p=0.765; large plastic: F(1,78)=2.05, p=0.156; large glass: F(1,78)=1, p=0.32).

In the two conditions that elicited the highest overall failure rate – small plastic and small glass conditions – the failure rate in subsequent trials did not decrease (the highest rate for small plastic: 50%, 4th trial, for small glass: 63%, 3rd trial, the lowest rate for small plastic: 0%, 1st trial, for small glass: 0%, 2nd, 8th and 9th trial ex aequo). For details see Supplementary Table 3.

**Supplementary Table 2.** Summary of the individual performance in each of the four conditions, including the number of correct trials (performance out of 10) and which trials subjects did not succeed on (trial no. of the error).

| **Subject** | **Performance out of 10** | | | | **Trial no. of the error** | | | |
| --- | --- | --- | --- | --- | --- | --- | --- | --- |
|  | **Small cylinder** | | **Large cylinder** | | **Small cylinder** | | **Large cylinder** | |
|  | **Plastic** | **Glass** | **Plastic** | **Glass** | **Plastic** | **Glass** | **Plastic** | **Glass** |
| **Ina** | 6 | 8 | 9 | 10 | 4,6,7,10 | 1,3 | 2 | - |
| **Filemon** | 5 | 9 | 10 | 10 | 3,4,8,9,10 | 3 | - | - |
| **Timmy** | 9 | 7 | 10 | 10 | 5 | 3,7 | - | - |
| **Stefan** | 7 | 9 | 9 | 10 | 2,7,8 | 9 | 10 | - |
| **Neffie** | 9 | 10 | 10 | 10 | 6 | - | - | - |
| **Lupus** | 8 | 8 | 10 | 10 | 8,10 | 3,6 | - | - |
| **Milton** | 9 | 7 | 10 | 10 | 4 | 4,7,10 | - | - |
| **Mira** | 6 | 9 | 10 | 9 | 2,4,6,9 | 3 | - | 10 |

# Experiment 2: Procedure

For details on the timing of all conditions see Supplementary Table 4.

**Supplementary Table 3.** The time elapsed (in days) since the first condition in Experiment 2.

| **Group** | **Name** | **All conditions** | | | |
| --- | --- | --- | --- | --- | --- |
| **1** | **All cats** | 0 | 1 | 2 | 3 |
| **2** | **Neffie** | 0 | 1 | 6 | 13 |
|  | **Lupus** | 0 | 1 | 20 | - |
|  | **Milton** | 0 | 1 | 20 | 22 |
|  | **Mira** | 0 | 7 | 9 | - |

# Experiment 2: Results

We used the analysis of variance for 2^3^ factorial design to estimate the effect of barrier length and goal distance on the success rate and confirmed the results in the best general linear model selection with the individual score as a response variable, and two predictor variables: barrier length and goal distance. The analysis revealed neither the interaction effect nor the main effect for length (*p* = 0.162). The main effect for size was significant (*p*<0.001). For details see Supplementary Figure 5.

**Supplementary Figure 2:** Main effect of goal distance on subjects’ average success rate (percentage of N=20 trials correct).

For details on the individual performance in Experiment 2 see Supplementary Table 6.

**Supplementary Table 4.** Summary of the individual performance in each of the six conditions, including the number of correct trials (performance out of 10) and which trials subjects did not succeed on (trial no. of the error).

| **Subject** | **Performance out of 10** | | | | | | **Trial no. of the error** | | | | | |
| --- | --- | --- | --- | --- | --- | --- | --- | --- | --- | --- | --- | --- |
|  | **Short barrier** | | | **Long barrier** | | | **Short barrier** | | | **Long barrier** | | |
|  | **0 cm** | **4.5 cm** | **9 cm** | **0 cm** | **4.5 cm** | **9 cm** | **0 cm** | **4.5 cm** | **9 cm** | **0 cm** | **4.5 cm** | **9 cm** |
| **Ina** | 4 | 9 | 10 | 5 | 10 | 10 | 1,2,3,4, 6, 8 | 3 | - | 4,5,7,8,10 | - | - |
| **Timmy** | 8 | 9 | 10 | 6 | 10 | 10 | 1,9 | 1 | - | 1,3,5,7 | - | - |
| **Stefan** | 5 | 10 | 10 | 3 | 7 | 10 | 2,3,4,5,6 | - | - | 1,2,4,5,8,9,10 | 3,5,7 | - |
| **Neffie** | 4 | 10 | 10 | 3 | 10 | 10 | 1,4,5,7,9,10 | - | - | 2,3,4,5,7,9,10 | - | - |
| **Lupus** | 5 | 10 | 10 | 5 | 10 | 10 | 1,2,3,4,5 | - | - | 1,2,3,4,7 | - | - |
| **Milton** | 9 | 9 | 10 | 6 | 10 | 10 | 4 | 2 | -- | 1,2,4,5 | - | - |
| **Mira** | 6 | 10 | 10 | 5 | 10 | 10 | 1,2,3,7 | - |  | 1,2,3,4,5 | - | - |

# Experiment 3: Procedure

For details on the timing of all conditions see Supplementary Table 7.

**Supplementary Table 5.** The time elapsed (in days) since the first condition in Experiment 3.

| **Group** | **All conditions** | | | |
| --- | --- | --- | --- | --- |
| **1** | 0 | 1 | 2 | 3 |
| **2** | 0 | 105 | - | - |

# Experiment 3: Results

We used the analysis of variance for 2^2^ factorial design to estimate the effect of barrier length and opening size on the success rate and confirmed the results in the best general linear model selection with the individual score as a response variable, and two predictor variables: barrier length and opening size. The analysis revealed neither the interaction effect nor the main effect for length (*p* = 0.8234). The main effect for size was significant (*p*<0.001). For details see Supplementary Figure 8.

**Supplementary Figure 3:** Main effect of opening size on subjects’ average success rate (percentage of N=20 trials correct).

For details on the individual performance in Experiment 3 see Supplementary Table 9.

**Supplementary Table 6:** Summary of the individual performance in each of the four conditions, including the number of correct trials (performance out of 10) and which trials subjects did not succeed on (trial no. of the error).

| **Subject** | **Performance out of 10** | | | | **Trial no. of the error** | | | |
| --- | --- | --- | --- | --- | --- | --- | --- | --- |
|  | **Small opening** | | **Large opening** | | **Small opening** | | **Large opening** | |
|  | **Short** | **Long** | **Short** | **Long** | **Short** | **Long** | **Short** | **Long** |
| **Ina** | 5 | 6 | 8 | 10 | 1,2,5,7,9 | 1,4,9,10 | 2,7 | - |
| **Timmy** | 9 | 9 | 10 | 10 | 2 | 3 | - | - |
| **Stefan** | 9 | 7 | 10 | 10 | 4 | 1,2,7 | - | - |
| **Neffie** | 9 | 10 | 10 | 10 | 10 | 10 | - | - |
| **Lupus** | 10 | 9 | 10 | 10 | - | 9 | - | - |
| **Milton** | 10 | 10 | 10 | 10 | - | 10 | - | - |
| **Mira** | 9 | 8 | 10 | 9 | 10 | 8 | - | 7 |

All statistical analyses were conducted in *R* (v.3.3.2, the R Foundation for Statistical Computing: http://www.R-project.org) using the packages car, lattice, gplots, glmulti and effects. Significance level was set at 0.05.
